# Approximate Solution for Electroosmotic Flow of Power-Law Fluids in a Planar Microchannel with Asymmetric Electrochemical Boundary Conditions

**DOI:** 10.3390/mi9060265

**Published:** 2018-05-28

**Authors:** WooSeok Choi, Sungchan Yun, Du-Soon Choi

**Affiliations:** Department of Mechanical Engineering, Korea National University of Transportation, Chungju 27469, Korea; w.choi@ut.ac.kr

**Keywords:** electroosmosis, power-law fluid, zeta potential, microchannel

## Abstract

Electroosmotic flow (EOF) is widely used in microfluidic systems and chemical analysis. It is driven by an electric force inside microchannel with highly charged boundary conditions. In practical applications, electrochemical boundary conditions are often inhomogeneous because different materials as walls are commonly utilized in routine fabrication methods. In the present study, we focus on the analytic solutions of the EOF generated in a planar microchannel with asymmetric electrochemical boundary conditions for non-Newtonian fluids. The velocity profile and flow rate are approximated by employing the power-law model of fluids in the Cauchy momentum equation. The hydrodynamic features of the EOF under asymmetric zeta potentials are scrutinized as a function of the fluid behavior index of the power-law fluid, thickness of Debye length, and zeta potential ratios between planes. The approximate solutions of the power-law model are comparable to the numerically obtained solutions when the Debye length is small and the fluid behavior index is close to unity. This study provides insights into the electrical control of non-Newtonian fluids, such as biological materials of blood, saliva, and DNA solution, in lab-on-a-chip devices.

## 1. Introduction

In the micro-scale, there have been intensive studies to adjust fluid movement using electrostatic force because pressure-driven flow is inefficient due to the extremely small hydraulic areas in the microchannel. Electroosmotic flow (EOF) is one of the well-known techniques in electrokinetics because it can efficiently drive fluids along microchannel devices, such as lab-on-a-chip [1,2,3,4]. Most studies on EOF are based on Newtonian fluids because most electrolytes or buffer solutions used in microfluidic devices are Newtonian. However, most biological fluids used in biochip, such as blood, saliva, and DNA solutions, are non-Newtonian fluids. As non-Newtonian fluids exhibit significantly different properties from Newtonian fluids, a model for non-Newtonian fluids should be established to predict proper EOF [5,6,7,8,9,10,11].

There are various constitutive models to analyze non-Newtonian fluids, such as power-law, Carreau, Phan–Thien–Tannar, and Oldroyd models. Among them, the power-law model (also known as the Ostwald–de Waele model) has become a convenient choice for its simplicity and adequateness in analyzing flow behavior. Therefore, there have been many studies that analyzed EOF using the power-law model [12,13,14,15,16,17,18,19,20]. Zhao et al. analyzed the behavior of EOF in a slit microchannel using power-law and obtained an approximated solution for shear stress and velocity distribution [12]. In addition, they presented the Smoluchowski slip velocity of the EOF in the microchannel using power-law and suggested an exact solution for the EOF of power-law fluids in the microchannel [14,16]. Tang et al. numerically analyzed the EOF of power-law fluids using the lattice Boltzmann method [13]. Vasu and De presented the mathematical model for the EOF of power-law fluids in a rectangular microchannel with a high zeta potential [15]. Babaie et al. and Hadigol et al. numerically analyzed the EOF of power-law fluids in a slit microchannel combined with a pressure gradient [17,18]. The aforementioned studies are based on EOF in a slit microchannel with symmetrical electrochemical boundary conditions assuming identical zeta potentials of the top and bottom walls. However, there are many cases where devices are made using different materials to simply fabricate a microchannel by using silicon dioxide (glass) as a base and polydimethylsiloxane (PDMS) as the top and side wall. When microchannels are fabricated using different materials, the zeta potentials are inhomogeneous, so asymmetric electrochemical boundary conditions should be introduced to reasonably predict EOF.

Studies on the EOF of non-Newtonian fluids with asymmetric zeta potentials are limited. Afonso et al. analyzed the EOF of non-Newtonian fluids in a microchannel using the simplified Phan–Thien–Tannar model and the finitely extensible nonlinear elastic model, Peterlin’s approximation [21]. Choi et al. obtained an analytical solution for the velocity profile of the EOF in the microchannel with asymmetric zeta potential using the Phan–Thien–Tannar model [22]. Qi and Ng investigated the EOF of a power-law fluid through a slit channel with an asymmetrically patterned wall [23]. Hadigol et al. presented the electroosmotic mixing of power-law fluid in a slit microchannel with nonuniform zeta potential distributions along the microchannel walls [24]. Recently, Choi et al. presented a numerical analysis for the electroosmotic velocity profiles of the power-law fluid in a rectangular microchannel with an asymmetric zeta potential using finite element analysis [25]. In these studies, it is difficult to understand intuitively how the parameters affect the velocity profile of the EOF because it is the result of numerical analysis or a very complicated form of expression.

In the present study, we generalize previous studies on power-law fluids and introduce bounding walls with different zeta potentials. Approximated solutions for the velocity profile and flow rate of the EOF of power-law fluids in a slit microchannel with asymmetric zeta potential were obtained and compared with previous numerical results. We report here a case of power-law fluids as a representative and comprehensive example.

## 2. Mathematical Formulation

**Figure 1:**
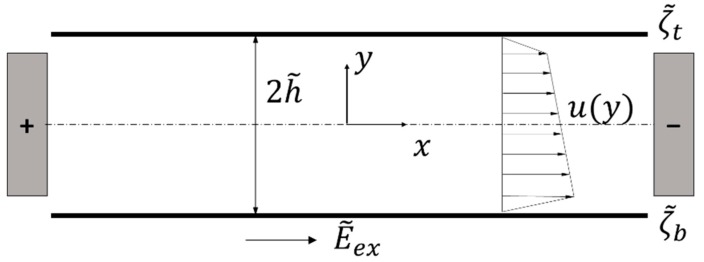
Schematic diagram of electroosmotic flow in a slit microchannel.

We consider a two-dimensional EOF, as shown in Figure 1, where a direct current of electric potential gradient ${\tilde{E}}_{ex}$ is applied to a power-law fluid with a constant density $\tilde{\rho }$ and electric permittivity $\tilde{\epsilon }$ in a microchannel with gap $2\tilde{h}$. The top and bottom boundaries bear electric charges upon contact with the fluid, quantified by zeta potentials ${\tilde{\zeta }}_{t}$ and ${\tilde{\zeta }}_{b}$, respectively. Thin nano-scale regions, called electrical double layers (EDLs), with excess ions are formed adjacent to the boundaries of the microchannel because of the zeta potential. The ions near the top and bottom EDLs are moved by the external electric field ${\tilde{E}}_{ex}$, and the movement of the ions generates the flow of fluid in the microchannel due to viscosity.

We describe this EOF theoretically by using a system of equation based on first principles and nondimensionalized using $\tilde{h}$, ${\tilde{E}}_{ex}\tilde{h}$, ${\tilde{u}}_{s}=-\tilde{\epsilon }{\tilde{\zeta }}_{b}{\tilde{E}}_{ex}/{\tilde{\mu }}_{0}$, and $\tilde{t}=\tilde{h}/{\tilde{u}}_{s}$ as the characteristic length, electric potential, velocity, and time, respectively, where ${\tilde{u}}_{s}$ and ${\tilde{\mu }}_{0}$ are the conventional Smoluchowski velocity and dynamic viscosity of Newtonian fluids and the superscript tilde denotes a dimensional form. The velocity field of the fluid in the microchannel is governed by the dimensionless continuity and Cauchy momentum equations, which are given as:

(1) ∇·u=0

(2) ReDuDt=−∇p+∇·τ+fe

where u is the velocity vector, *p* is the pressure, τ is the stress tensor, and $f_{e}$ is the body force. We consider the unidirectional flow that can be represented as $u=u\left(y\right)e_{x}$, where *u* is the *x-*component of velocity and $e_{x}$ is the unit vector along the *x*-direction. The body force $f_{e}$ acting on the fluid is defined as the product of the free charge density and the external electric field, and the density of the charge can be obtained by Poisson’s equation. The fluid in the microchannel is assumed to be *x*-direction unidirectional flow, and the shear stress of the power-law fluid is defined as [26]:

(3) $$\tau =m{\left|\frac{\partial u}{\partial y}\right|}^{n-1}\frac{\partial u}{\partial y}$$

where u is the *x*-directional velocity of the fluid; m is the flow consistency index, which is nondimensionalized using ${\tilde{\mu }}_{0}{\tilde{h}}^{n-1}/{\tilde{u}}_{s}^{n-1}$; and n is the flow behavior index. If the pressure gradient is neglected, Equation (2) can be expressed as:

(4) $$0=nm{\left|\frac{\partial u}{\partial y}\right|}^{n-1}\frac{\partial^{2}u}{\partial y^{2}}-\frac{1}{E_{R}}\frac{\partial \phi }{\partial x}\frac{\partial^{2}\phi }{\partial y^{2}}$$

where ϕ is the nondimensional electric potential and $E_{R}$ is a dimensionless parameter indicating the ratio of zeta potential to external potential, $E_{R}={\tilde{\zeta }}_{b}/{\tilde{E}}_{ex}\tilde{h}$. The details on the dimensionless form are described in Appendix A. The total electric potential can be represented as ϕ(x,y)=−x+φ(y), where −x and φ(y) are the electric potential due to an external electric field and zeta potentials, respectively. Here the Debye length (${\tilde{\lambda }}_{D}$), a measure of the EDL thickness, is expressed as ${\tilde{\lambda }}_{D}={\left(\tilde{\epsilon }{\tilde{k}}_{B}\tilde{T}/2{\tilde{e}}^{2}{\tilde{z}}^{2}{\tilde{N}}_{A}{\tilde{c}}_{0}\right)}^{1/2}$ for an aqueous solution of a symmetrical electrolyte, where ${\tilde{k}}_{B}$ is a Boltzmann constant, $\tilde{T}$ is the absolute temperature, $\tilde{e}$ is the elementary charge density, $\tilde{z}$ is the charge number of ions, ${\tilde{N}}_{A}$ is the Avogadro’s number, and ${\tilde{c}}_{0}$ is the mole concentration ($\mathrm{mol}/m^{3}$). If the Debye length ${\tilde{\lambda }}_{D}$ is extremely small compared with the microchannel thickness $2\tilde{h}$, the electric potential due to the zeta potential can be described by the Poisson–Boltzmann equation, which can be linearized with the Debye–Hückel approximation as:

(5) $$\frac{\partial^{2}\varphi }{\partial y^{2}}=\frac{\varphi }{L_{D}^{2}}$$

where $L_{D}={\tilde{\lambda }}_{D}/\tilde{h}$ is the nondimensional Debye length. If the operation 〈·〉 is defined as ${\langle a\rangle }^{b}=a{\left|a\right|}^{b-1}$, by integrating Equation (4), the approximate velocity gradient is obtained as:

(6) $$\frac{\partial u}{\partial y}=-{\langle \chi \rangle }^{1/n}{\langle Z_{t}\exp\left(\frac{y-1}{L_{D}}\right)-Z_{b}\exp\left(-\frac{y+1}{L_{D}}\right)+\alpha \rangle }^{1/n}$$

where $\chi =1/\left(mE_{R}L_{D}\right)$, α is an unknown constant, and $Z_{t}$ and $Z_{b}$ are the nondimensional zeta potential of the top and bottom walls, respectively. The details of the equation are described in Appendix B. In Equation (6), the term $\exp\left(\left(y-1\right)/L_{D}\right)Z_{t}$ can be interpreted as the effect of $Z_{t}$ on the flow near the top boundary, the term $-\exp\left(-\left(y+1\right)/L_{D}\right)Z_{b}$ is the component generated by the movement of ions near the bottom wall, and α is caused by the flow rate difference near the top and bottom walls. The first term is almost zero near the bottom wall, and the second term is near the top wall. Therefore, the velocity gradient can be approximated by dividing it into two sections as:

(i) 0≤y≤1

(7) $$\frac{\partial u_{t}}{\partial y}=-{\langle \chi \rangle }^{1/n}{\langle \exp\left\{\frac{y-1}{L_{D}}\right\}Z_{t}+\alpha \rangle }^{1/n}$$

(ii) −1≤y<0

(8) $$\frac{\partial u_{b}}{\partial y}=-{\langle \chi \rangle }^{1/n}{\langle -\exp\left\{-\frac{y+1}{L_{D}}\right\}Z_{b}+\alpha \rangle }^{1/n}$$

where $u_{t}$ and $u_{b}$ are the velocity at the top and bottom halves of the channel, respectively. If Equations (7) and (8) are integrated as it is, the velocity is represented as a hypergeometric function, which is an infinite series, so intuitive understanding of the velocity profile is very difficult. In the top half of the channel, the term $\exp\left(\left(y-1\right)/L_{D}\right)Z_{t}$ is dominant near the top wall (y≈1), and α is dominant near the center line of the microchannel (y≈0). If the term $\exp\left(\left(y-1\right)/L_{D}\right)Z_{t}$. is dominant, the velocity gradient in Equation (7) will be approximated as $\chi^{1/n}\exp\left(\left(y-1\right)/L_{D}\right)Z_{t}{}^{1/n}$ because extra terms are very small, and vice versa. Therefore, the velocity gradient from Equation (7) can be approximated as:

(9) $$\frac{\partial u_{t}}{\partial y}\approx -{\langle \chi \rangle }^{1/n}\left[\exp\left\{\frac{1}{n}\frac{y-1}{L_{D}}\right\}{\langle Z_{t}\rangle }^{1/n}+{\langle \alpha \rangle }^{1/n}\right]$$

Although the prosed approximation is inaccurate between the regions where the size of the two terms is similar, the range of this inaccurate region is very limited because the term $\exp\left(\left(y-1\right)/L_{D}\right)Z_{t}$ changes exponentially. Therefore, the effect of the approximation error on the overall velocity profile will be small. If the fluid in the bottom half of the channel is applied in the same way, Equation (8) can be approximated as:

(10) $$\frac{\partial u_{b}}{\partial y}\approx -{\langle \chi \rangle }^{1/n}\left[-\exp\left\{-\frac{1}{n}\frac{y+1}{L_{D}}\right\}{\langle Z_{b}\rangle }^{1/n}+{\langle \alpha \rangle }^{1/n}\right]$$

The velocity obtained by integrating Equations (9) and (10) is expressed as:

(11) $$u_{t}=-{\langle \chi \rangle }^{1/n}{\langle Z_{t}\rangle }^{1/n}nL_{D}\exp\left\{\frac{1}{n}\frac{y-1}{L_{D}}\right\}+{\langle \chi \rangle }^{1/n}\alpha^{1/n}y+\beta_{t}$$

(12) $$u_{b}=-{\langle \chi \rangle }^{1/n}{\langle Z_{b}\rangle }^{1/n}nL_{D}\exp\left\{-\frac{1}{n}\frac{y+1}{L_{D}}\right\}+{\langle \chi \rangle }^{1/n}\alpha^{1/n}y+\beta_{b}$$

where $\beta_{t}$ and $\beta_{b}$ are integral constants. At the center of the microchannel (y=0), the streamwise component of the velocity must be continuous:

(13) $$u_{t}\left(0\right)=u_{b}\left(0\right)$$

As $L_{D}\ll 1$, the term $\exp\left(-1/(nL_{D}\right))$ converges to zero at y=0. Thus, the integral constants $\beta_{t}$ and $\beta_{b}$ have the same value ($\beta \approx \beta_{t}\approx \beta_{b}$). In Equations (11) and (12), the terms ${\left(\chi \alpha \right)}^{1/n}+\beta$ are common, the term $\exp\left\{\left(y-1\right)/\left(nL_{D}\right)\right\}$ converges zero at y≤0, and the term $\exp\left\{-\left(y+1\right)/\left(nL_{D}\right)\right\}$ converges zero at y≥0. The velocity can be expressed as:

(14) $$u=-{\langle \chi \rangle }^{1/n}\left[nL_{D}\left\{{\langle Z_{t}\rangle }^{1/n}\exp\left(\frac{y-1}{nL_{D}}\right)+{\langle Z_{b}\rangle }^{1/n}\exp\left(-\frac{y+1}{nL_{D}}\right)\right\}+\alpha^{1/n}y+c\right]$$

where $c=\beta /\chi^{1/n}$. On the top and bottom walls, no-slip boundary conditions $u_{t}=0$ at y=1 and $u_{b}=0$ at y=−1 are imposed. The above system yields the constant as:

(15) $$\alpha^{1/n}=-\frac{nL_{D}}{2}\left\{{\langle Z_{t}\rangle }^{1/n}-{\langle Z_{b}\rangle }^{1/n}\right\}$$

(16) $$c=-\frac{1}{2}nL_{D}\left\{{\langle Z_{t}\rangle }^{1/n}+{\langle Z_{b}\rangle }^{1/n}\right\}$$

In this study, potential ratio $E_{R}$ and the dimensionless zeta potential of the bottom wall $Z_{b}$ have the same value ($E_{R}=Z_{b}={\tilde{\zeta }}_{b}/{\tilde{E}}_{ex}\tilde{h}$), and thus, the velocity profile can be simplified as:

(17) $$u=-\frac{nL_{D}}{{\left(mL_{D}\right)}^{1/n}}\left\{{\langle Z_{R}\rangle }^{1/n}\exp\left(\frac{y-1}{nL_{D}}\right)+\exp\left(-\frac{y+1}{nL_{D}}\right)-\frac{y}{2}\left({\langle Z_{R}\rangle }^{1/n}-1\right)-\frac{1}{2}\left({\langle Z_{R}\rangle }^{1/n}+1\right)\right\}$$

where $Z_{R}$ is the zeta potential ratio $Z_{R}=Z_{t}/Z_{b}$. As we know, the magnitude of zeta potential is independent of the velocity of EOF, and the flow consistency index m affects the magnitude of EOF but does not affect the velocity profiles. The dimensionless flow rate Q can be obtained by a straightforward integration of the velocity profile across the depth of the microchannel (−1≤y≤1) as:

(18) $$Q=\int_{-1}^{1}udy=\chi^{1/n}nL_{D}\left(nL_{D}-1\right)\left\{{\langle Z_{t}\rangle }^{1/n}+{\langle Z_{b}\rangle }^{1/n}\right\}=-\frac{nL_{D}}{{\left(mL_{D}\right)}^{1/n}}\left(nL_{D}-1\right)\left\{{\langle Z_{R}\rangle }^{1/n}+1\right\}$$

The parameters used in this study are the zeta potential ratio, which ranges from −0.5 to 1.5; the fluid behavior index (n), which ranges from 0.8 to 1.2; and the dimensionless Debye length ($L_{D}$), which ranges from 0.001 to 0.1.

## 3. Discussions

In this study, the accuracy of the approximate velocity profile is compared with the results of numerical analysis in a two-dimensional microchannel based on previous work from our group. For comparison, the present results are plotted by lines, and the numerical results are plotted by symbols.

**Figure 2:**
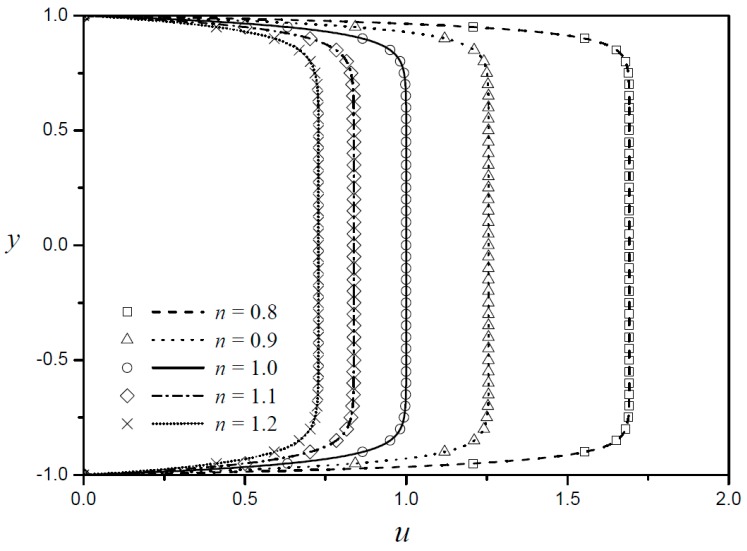
Velocity profiles obtained by the present result and numerical analysis for five different fluid behavior indexes for $L_{D}=0.05$ and $Z_{R}=1$.

When both the top and bottom boundaries are made of the same materials ($Z_{R}=1$), α becomes zero, the assumption used in Equations (9) and (10) are ignored, and an accurate expression can be obtained. Figure 2 shows such cases for five different fluid behavior indexes. The present results (lines) and those by numerical analysis (symbols) are seen to coincide exactly for all five cases. For a Newtonian fluid (n=1), the characteristic plug-type EOF with Helmoholtz–Smoluchowski velocity in the core part of the flow and a large velocity gradient in the thin boundary regions are observed. The shear thinning fluid (smaller fluid behavior index) is less viscous with increasing velocity gradient, and thus the EOF is enhanced as the fluid behavior index decreases.

**Figure 3:**
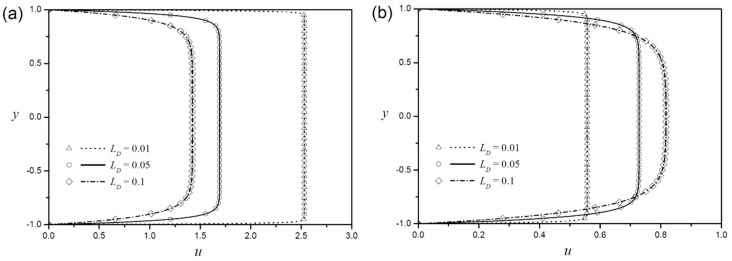
Velocity profiles with symmetric zeta potentials according to different Debye lengths for (**a**) shear-thinning fluid (n=0.8) and (**b**) shear-thickening fluid (n=1.2 ).

The change of Debye length $L_{D}$ in a Newtonian fluid affects the velocity profile near the boundary, but the velocity of the core part (Smoluchowski velocity) is identical [1,2,3]. Figure 3 shows the velocity profile with the change in Debye length in the power-law fluid. The velocity profiles for a shear-thinning fluid (n=0.8) are plotted in Figure 3a, and those for a shear-thickening fluid (n=1.2) are plotted in Figure 3b. In non-Newtonian fluids, the velocity gradient is concentrated in a narrow region near the boundaries, and the velocity profiles approach the plug flow as the Debye length decreases, which is similar to the Newtonian fluid. However, these results also show a meaningful difference between non-Newtonian and Newtonian fluids at the EOF. Unlike the Newtonian fluid, the non-Newtonian fluid changes velocity at the core part with the change in Debye length. Interestingly, the effect of Debye length on the velocity at the core region is reversed in shear thinning and shear thickening fluids. In a shear-thinning fluid, the velocity at the core is more enhanced, as the Debye length decreases, whereas the shear-thickening fluid exhibits the opposite effect.

**Figure 4:**
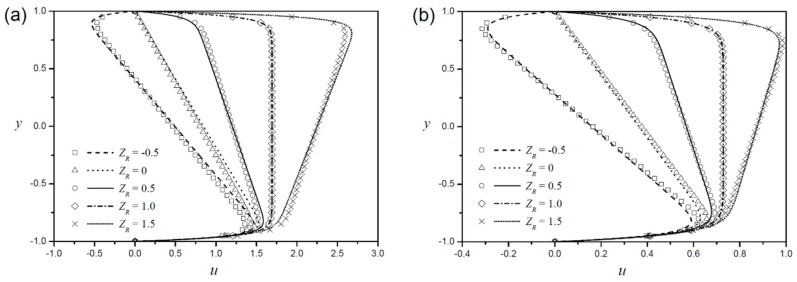
Velocity profiles according to different zeta potential ratios with a fixed Debye length ($L_{D}=0.05$) for (**a**) shear thinning fluid (n=0.8 ) and (**b**) shear thickening fluid (n=1.2 ).

Figure 4 shows the velocity profiles when the top and bottom boundaries are made of different materials and thus of different zeta potentials ($Z_{R}\neq 1$). The symmetric case ($Z_{R}=1$) is included in the figure as a reference. If the zeta potential at the top boundary is higher (lower) than that at the bottom, the flow near the top boundary is more (less) enhanced than the flow near the bottom, and the velocity gradient is enhanced at the core unlike the symmetric case. At the asymmetric case ($Z_{R}\neq 1$), the constant α of Equation (15) is non-zero, which is different from the symmetric case, thereby resulting in errors due to the assumptions in Equations (9) and (10). As shown in the figure, the errors in the approximate solutions are affordable, so the assumptions used in Equations (9) and (10) are available. When the zeta potentials on the top and bottom boundaries have opposite signs ($Z_{R}<0$), the ions adjacent the top boundary are subjected to forces in the opposite direction to the ions adjacent the bottom boundary, so EOF will be generated as seen in the figure. The larger the difference between the zeta potential of each wall, the greater the error in the approximate solutions. Under the same zeta potential difference, the sign of the error of the shear-thinning fluid and that of the shear-thickening fluid are opposite.

**Figure 5:**
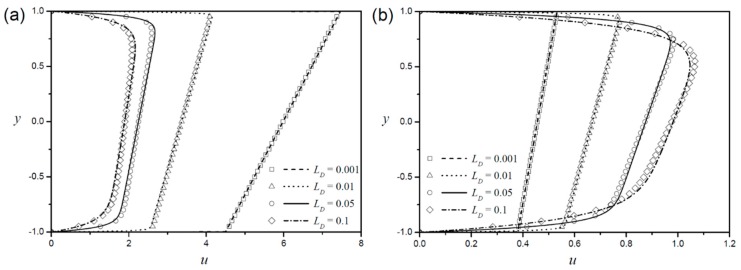
Velocity profiles according to the Debye length with a fixed zeta potential ratios ($Z_{R}=1.5$) for (**a**) shear thinning fluid (n=0.8 ) and (**b**) shear thickening fluid (n=1.2 ).

**Figure 6:**
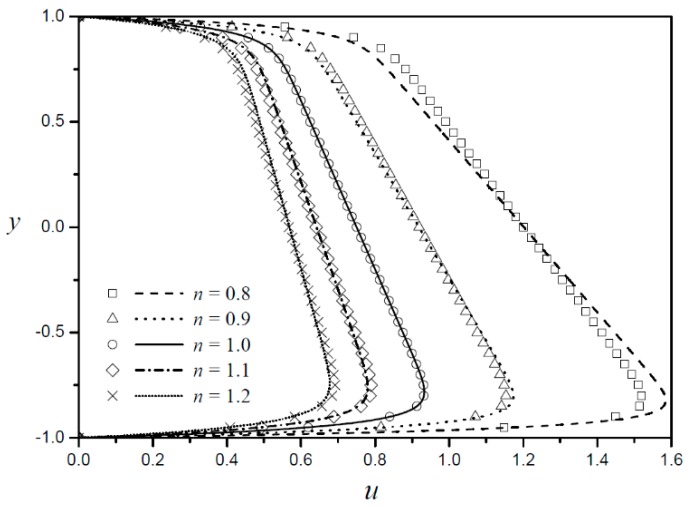
Velocity profiles according to the fluid behavior index with fixed zeta potential ratios ($Z_{R}=0.5$) and Debye length ($L_{D}=0.05$ ).

Figure 5 shows the velocity profiles according to the Debye length with asymmetric zeta potentials for the shear-thinning fluid (Figure 5a) and shear-thickening fluid (Figure 5b). The errors between the approximated solution and numerical analysis decreases as the Debye length decreases. The Debye length used in this study is generally larger than the Debye length used in the actual EOF to observe the velocity profile near the boundaries. If a univalent electrolyte with 1 mM ion concentration is used for a microchannel of 20 µm height, the dimensionless Debye length $L_{D}\approx 0.001$, which is much smaller than the Debye length used in this study. Figure 6 shows the velocity profiles for five different fluid behavior indexes with fixed zeta potential ratio ($Z_{R}=0.5$) and Debye length ($L_{D}=0.05$). In this figure, the approximated solution and numerical results coincide at the Newtonian fluids, and the error increases as the shear thinning or thickening properties become stronger. The percent difference between the maximum velocities form the approximate solution and numerical results are described in the Supplementary Materials (Tables S1 and S2).

**Figure 7:**
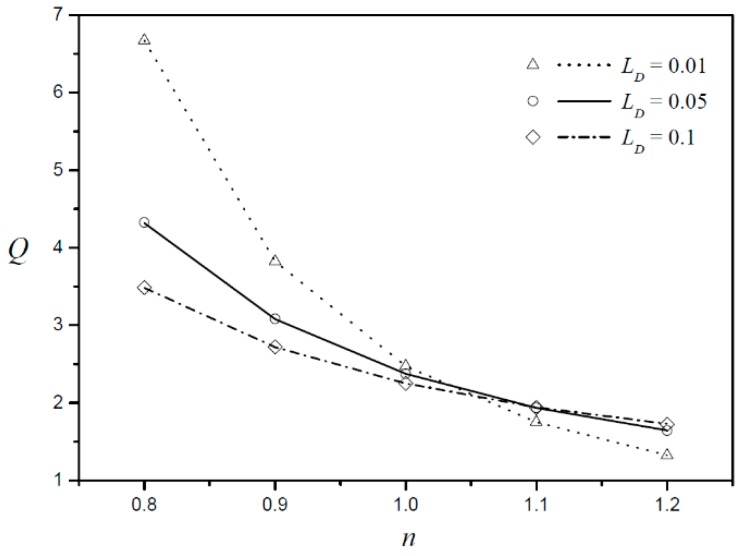
Dimensionless flow rate according to fluid behavior index (n) for different Debye lengths ($L_{D}$) with a fixed zeta potential ratio ($Z_{R}=1.5$ ).

The EOF can be examined more quantitatively by studying the volumetric flow rate, which can be obtained by integrating the velocity profiles across the depth of the microchannel (−1≤y≤1). Figure 7 shows the volumetric flow rate according to the fluid behavior index for three different Debye lengths with a fixed zeta potential ratio ($Z_{R}=1.5$). Given that the sign of error at the approximate solution in the top half of the channel is opposite that in the bottom half of the channel, these errors are canceled out in the integration to obtain the flow rate. Therefore, the flow rate of the approximate equation is in good agreement with the numerical results. Table 1 shows the flow rate error $Q_{er}$ defined as $Q_{er}=\left|Q_{as}-Q_{na}\right|/Q_{na}\times 100$, where $Q_{as}$ is the flow rate obtained from the approximate solution and $Q_{na}$ is the flow rate obtained from the numerical analysis. The largest error rate is approximately 0.2%, so the flow rate of the approximate equation is fairly accurate. The volumetric flow rate decreases with increasing fluid behavior index. The smaller the Debye length is, the larger the flow rate decreases as the fluid behavior index changes.

## 4. Conclusions

In this study, the approximate solution for fully developed two-dimensional steady unidirectional EOFs of power-law fluids with different zeta potentials on the top and bottom boundaries was derived. The approximate solution was compared with the numerical analysis results. The approximate solution is identical to the numerical results in the case of the Newtonian fluid (n=1) or the zeta potentials of the top and bottom boundaries being equal. The approximate solution is similar to the numerical results as the Debye length becomes smaller or the fluid behavior index is closer to unity. Given that the velocity errors obtained from the upper and lower halves of the channel cancel each other out, the volumetric flow rate obtained by the approximate solution is accurate. In many cases, the microchannels are made up of different materials, and the Debye length is very small. Therefore, this approximate solution helps in efficiently designing a microfluidic system using EOF without numerical analysis.

## Figures and Tables

**Table 1:** Flow rate error (%) between approximate solution and numerical analysis at $Z_{R}=1.5$.

n	$L_{D}=0.01$	$L_{D}=0.05$	$L_{D}=0.1$
0.8	0.012	0.090	0.135
0.9	0.013	0.071	0.096
1.0	0	0	0
1.1	0.023	0.088	0.108
1.2	0.038	0.171	0.220

## Supplementary Materials

The following are available online at http://www.mdpi.com/2072-666X/9/6/265/s1. Table S1: Flow rate error (%) between approximate solution and numerical analysis at $Z_{R}=1.5$, Table S2: Flow rate error (%) between approximate solution and numerical analysis at $Z_{R}=0.5$.

## Appendix A

The Cauchy momentum equation of the power-law fluid can be non-dimensionalized in the following ways:

(A1) $$\tilde{\rho }\frac{D\tilde{u}}{D\tilde{t}}=-\tilde{\nabla }\tilde{p}+\tilde{\nabla }\cdot \tilde{\tau }+{\tilde{f}}_{e}$$

(A2) $$\tilde{\rho }\frac{{\tilde{u}}_{s}^{2}}{\tilde{h}}\frac{Du}{Dt}=-\frac{{\tilde{\mu }}_{0}{\tilde{u}}_{s}}{{\tilde{h}}^{2}}\nabla p+\frac{{\tilde{\mu }}_{0}{\tilde{u}}_{s}}{{\tilde{h}}^{2}}\nabla \cdot \tau +\frac{\tilde{\epsilon }{\tilde{E}}_{ex}^{2}}{\tilde{h}}\nabla \phi \nabla^{2}\phi$$

(A3) $$\tilde{\rho }\frac{{\tilde{u}}_{s}\tilde{h}}{{\tilde{\mu }}_{0}}\frac{Du}{Dt}=-\nabla p+\nabla \cdot \tau +\frac{\tilde{\epsilon }{\tilde{E}}_{ex}{\tilde{\zeta }}_{b}}{{\tilde{\mu }}_{0}}\frac{1}{{\tilde{u}}_{s}}\frac{{\tilde{E}}_{ex}\tilde{h}}{{\tilde{\zeta }}_{b}}\nabla \phi \nabla^{2}\phi$$

(A4) ReDuDt=−∇p+∇·τ−1ER∇ϕ∇2ϕ

where the superscript tilde denotes a dimensional form.

## Appendix B

Zeta potential conditions of $Z_{t}$ and $Z_{b}$ are imposed on the top (y=1) and bottom (y=−1) boundaries, respectively, and the dimensionless electric potential due to the zeta potential can be obtained from Equation (5).

(A5) $$\varphi =\frac{Z_{t}+Z_{b}}{2\cosh\left(1/L_{D}\right)}\cosh\left(\frac{y}{L_{D}}\right)+\frac{Z_{t}-Z_{b}}{2\sinh\left(1/L_{D}\right)}\sinh\left(\frac{y}{L_{D}}\right)$$

Given that Debye length $L_{D}\ll 1$, it can be assumed as:

(A6) $$\cosh\left(\frac{1}{L_{D}}\right)\approx \sinh\left(\frac{1}{L_{D}}\right)\approx \frac{1}{2}\exp\left(\frac{1}{L_{D}}\right)$$

The dimensionless velocity gradient equation (Equation (6) can be obtained in the following ways:

(A7) $$nm{\left|\frac{\partial u}{\partial y}\right|}^{n-1}\frac{\partial^{2}u}{\partial y^{2}}=-\frac{1}{E_{R}L_{D}^{2}}\left\{Z_{t}\exp\left(\frac{y-1}{L_{D}}\right)-Z_{b}\exp\left(-\frac{y+1}{L_{D}}\right)\right\}$$

(i) ∂u/∂y>0

(A8) $$m{\left(\frac{\partial u}{\partial y}\right)}^{n}=-\frac{1}{E_{R}L_{D}}\left\{Z_{t}\exp\left(\frac{y-1}{L_{D}}\right)-Z_{b}\exp\left(-\frac{y+1}{L_{D}}\right)+\alpha \right\}$$

(ii) ∂u/∂y<0

(A9) $$-m{\left(-\frac{\partial u}{\partial y}\right)}^{n}=-\frac{1}{E_{R}L_{D}}\left\{Z_{t}\exp\left(\frac{y-1}{L_{D}}\right)-Z_{b}\exp\left(-\frac{y+1}{L_{D}}\right)+\alpha \right\}$$

where α is the integral constant. As the velocity gradient ∂u/∂y should be a real number, the velocity gradient obtained from Equations (A8) and (A9) can be expressed as:

(i) ∂u/∂y>0, $E_{R}<0$
  (A10) $$\frac{\partial u}{\partial y}={\left(-\frac{1}{mE_{R}L_{D}}\right)}^{1/n}{\left[Z_{t}\exp\left(\frac{y-1}{L_{D}}\right)-Z_{b}\exp\left(-\frac{y+1}{L_{D}}\right)+\alpha \right]}^{1/n}$$
(ii) ∂u/∂y>0, $E_{R}>0$
  (A11) $$\frac{\partial u}{\partial y}={\left(\frac{1}{mE_{R}L_{D}}\right)}^{1/n}{\left[-\left\{Z_{t}\exp\left(\frac{y-1}{L_{D}}\right)-Z_{b}\exp\left(-\frac{y+1}{L_{D}}\right)+\alpha \right\}\right]}^{1/n}$$
(iii) ∂u/∂y<0, $E_{R}<0$
  (A12) $$\frac{\partial u}{\partial y}=-{\left(-\frac{1}{mE_{R}L_{D}}\right)}^{1/n}{\left[-\left\{Z_{t}\exp\left(\frac{y-1}{L_{D}}\right)-Z_{b}\exp\left(-\frac{y+1}{L_{D}}\right)+\alpha \right\}\right]}^{1/n}$$
(iv) ∂u/∂y<0, $E_{R}>0$
  (A13) $$\frac{\partial u}{\partial y}=-{\left(\frac{1}{mE_{R}L_{D}}\right)}^{1/n}{\left[Z_{t}\exp\left(\frac{y-1}{L_{D}}\right)-Z_{b}\exp\left(-\frac{y+1}{L_{D}}\right)+\alpha \right]}^{1/n}$$

If the operation 〈·〉 is defined as $a^{b}=a{\left|a\right|}^{b-1}$, combining Equations (A10) and (A11), the velocity gradient can be expressed as:

(A14) $$\frac{\partial u}{\partial y}=-{\langle \frac{1}{mE_{R}L_{D}}\rangle }^{1/n}{\langle Z_{t}\exp\left(\frac{y-1}{L_{D}}\right)-Z_{b}\exp\left(-\frac{y+1}{L_{D}}\right)+\alpha \rangle }^{1/n}$$
