# Supplementary material for: Approximate Solution for Electroosmotic Flow of Power-Law Fluids in a Planar Microchannel with Asymmetric Electrochemical Boundary Conditions
Source: Micromachines (Basel). 2018 May 28;9(6):265. doi: 10.3390/mi9060265 (PMC6187346; doi:10.3390/mi9060265)
Supplement: Supplementary file 1 [file micromachines-09-00265-s001.pdf]

Supplementary Materials: Approximate Solution for Electroosmotic Flow of Power-Law Fluids in a Planar Microchannel with Asymmetric Electrochemical Boundary Conditions

WooSeok Choi, Sungchan Yun * and Du-Soon Choi *

Maximum Velocity Differences

Table S1 and S2 shows the maximum velocity error u_{er} defined as $u_{er} = (u_{ma} - u_{mn})/u_{mn} \times 100$, where u_{ma} is the maximum velocity obtained from the approximate solution and u_{mn} is the maximum velocity obtained from the numerical analysis for various fluid behavior index and Debye length with a fixed zeta potential ratio.

Table S1. Flow rate error (%) between approximate solution and numerical analysis at Z_R = 1.5.

Fluid Behavior Index (n)	$L_D = 0.001$	$L_D = 0.01$	$L_D = 0.05$	$L_D = 0.1$
1.2	−0.13	−0.55	−1.50	−1.86
1.1	−0.09	−0.34	−0.89	−1.09
1.0	−0.04	−0.01	0.00	−0.01
0.9	0.07	0.53	1.27	1.55
0.8	0.28	1.43	3.17	3.81

Table S2. Flow rate error (%) between approximate solution and numerical analysis at Z_R = 0.5.

Fluid Behavior Index (n)	$L_D = 0.001$	$L_D = 0.01$	$L_D = 0.05$	$L_D = 0.1$
1.2	−0.15	−0.77	−2.07	−2.71
1.1	−0.10	−0.46	−1.21	−1.57
1.0	−0.01	−0.01	−0.01	0.00
0.9	0.13	0.70	1.71	2.19
0.8	0.42	1.86	4.24	5.33
